# An environmental habitat gradient and within-habitat segregation enable co-existence of ecologically similar bird species

**DOI:** 10.1098/rspb.2023.0467

**Published:** 2023-08-30

**Authors:** Samuel Ayebare, Jeffrey W. Doser, Andrew J. Plumptre, Isaiah Owiunji, Hamlet Mugabe, Elise F. Zipkin

**Affiliations:** ^1^ Department of Integrative Biology, Michigan State University, East Lansing, MI 48824, USA; ^2^ Ecology, Evolution, and Behavior Program, Michigan State University, East Lansing, MI 48824, USA; ^3^ KBA Secretariat, c/o BirdLife International, David Attenborough Building, Pembroke Street, Cambridge CB2 3QZ, UK; ^4^ Conservation Science Group, Zoology Department, Cambridge University, Pembroke Street, Cambridge CB2 3QZ, UK; ^5^ Kabale University, PO Box 317, Kabale, Uganda; ^6^ Wildlife Conservation Society (WCS), Uganda Programme, PO Box 7487, Kampala, Uganda

**Keywords:** coexistence, habitat partitioning, elevation gradient, Grinnelian niche, Eltonian niche, Albertine rift

## Abstract

Niche theory predicts that ecologically similar species can coexist through multidimensional niche partitioning. However, owing to the challenges of accounting for both abiotic and biotic processes in ecological niche modelling, the underlying mechanisms that facilitate coexistence of competing species are poorly understood. In this study, we evaluated potential mechanisms underlying the coexistence of ecologically similar bird species in a biodiversity-rich transboundary montane forest in east-central Africa by computing niche overlap indices along an environmental elevation gradient, diet, forest strata, activity patterns and within-habitat segregation across horizontal space. We found strong support for abiotic environmental habitat niche partitioning, with 55% of species pairs having separate elevation niches. For the remaining species pairs that exhibited similar elevation niches, we found that within-habitat segregation across horizontal space and to a lesser extent vertical forest strata provided the most likely mechanisms of species coexistence. Coexistence of ecologically similar species within a highly diverse montane forest was determined primarily by abiotic factors (e.g. environmental elevation gradient) that characterize the Grinnellian niche and secondarily by biotic factors (e.g. vertical and horizontal segregation within habitats) that describe the Eltonian niche. Thus, partitioning across multiple levels of spatial organization is a key mechanism of coexistence in diverse communities.

## 1. Introduction

Species coexistence patterns are a function of abiotic factors (e.g. climate, elevation, soil), biotic processes (e.g. competition, predation, mutualism) and dispersal filters (e.g. geographical barriers [1]). At a given location, ecologically similar species (here defined as potentially competing sympatric species belonging to the same family) may both be present or absent, or only one species may be present [2,3]. Species coexistence patterns (i.e. co-occurrence or co-abundance) measured at a fine scale (i.e. sampling point) provide insights into potential mechanisms enabling coexistence of ecologically similar species at a broad scale (e.g. study area). Coexistence patterns of similar species could be owing to shared environmental resources [4], interspecific interactions [5,6], character displacement [7], or chance [8]. These abiotic and biotic factors and processes underlying coexistence patterns are expressed via multiple mechanisms (e.g. spatial, temporal, diet, foraging behaviour) resulting in niche differentiation. While a variety of approaches exist to study species coexistence patterns (e.g. [9]), multi-species distribution models can be particularly useful for the analysis of large communities across broad spatial extents [10]. However, owing to the challenges of accounting for both abiotic and biotic processes in species distribution models, the underlying mechanisms that facilitate coexistence of potentially competing species are poorly understood [11].

Characterizing a species ecological niche typically follows either the Grinnelian approach that focuses on the abiotic environment and/or the Eltonian approach that focuses on the biotic environment [12,13]. Niche estimation approaches focusing on the abiotic environment have been widely applied [14,15], largely because it is easier to characterize species abiotic habitat conditions than measuring biotic processes, particularly using only observational datasets. However, incorporating biotic processes into species distribution models is of broad interest [11], owing to hypothesized benefits of improved characterization of ecological niches and a potential understanding of species coexistence mechanisms. Recent studies have attempted to account for biotic processes within species distribution models with a variety of approaches, including using potential competitor species as a predictor variable [4,16], surrogate variables that represent biotic interaction gradients [10], and simultaneously estimating the effects of abiotic factors and correlations of species occurrences or abundances [17]. Despite these methodological advances, two outstanding questions in ecological niche modelling are: (i) what are the underlying mechanisms that enable coexistence of ecologically similar species, and (ii) how do these mechanisms explain interspecific associations, either positive or negative? To answer these questions, we examine a diverse community of tropical birds and use competition theory, which predicts that for two closely related species to coexist they must differ in the degree of resource use along at least one niche dimension [2].

Birds are an excellent taxon to explore these fundamental questions of coexistence as they partition their niches along several measurable gradients via multiple mechanisms [18–20] that can be categorized as Grinellian (i.e. abiotic) or Eltonian (i.e. biotic) processes [12]. Grinellian variables are not affected by the presence of the target species, operate at broad scales, and are density independent. Alternatively, Eltonian variables are affected by the presence of the species, operate at fine scales, and are density dependent. Ecologically similar bird species could partition their niches through attributes that characterize the Eltonian niche such as vertical stratification of feeding zones [21], variation in activity pattern [22], differences in diet and foraging behaviour [23], interspecific territoriality [19,24], body size variation [25] and within-habitat segregation [26], and/or through factors that characterize the Grinellian niche such as climate and habitat variables [27].

In this study, we assessed potential mechanisms underlying the coexistence of ecologically similar bird species by computing niche overlap indices along abiotic (environmental elevation gradient) and biotic (diet, vertical foraging strata, activity patterns and within-habitat segregation across horizontal space) factors. Niche overlap indices measure the extent to which co-occurring species use the same resources in niche space. As such, the degree of niche overlap along different niche dimensions provides a mechanistic understanding of community structuring [18,28]. Along a given resource gradient, ecologically similar species can show no overlap, partial overlap or complete overlap [29]. Our specific objectives were: (i) to assess niche overlap along an elevation environmental gradient, an abiotic variable, and then (ii) determine whether the observed co-abundance patterns after accounting for this environmental variation could be attributed to niche partitioning along biotic variables.

We developed a hierarchical community model [30,31] to estimate co-abundance variation, and niche overlap indices among ecologically similar bird species along an elevation gradient in the Albertine Rift ecoregion in east-central Africa. The Albertine Rift is a biodiversity hotspot [32,33] supporting more bird species than elsewhere on the African continent [34], and has been designated as a globally important ecoregion for bird conservation [35]. Our study examines factors and processes that determine the distribution and abundance of birds in this highly diverse but poorly studied area and improves our understanding of coexistence mechanisms of potentially competing species.

## 2. Methods

### (a) Study area

**Figure 1.**
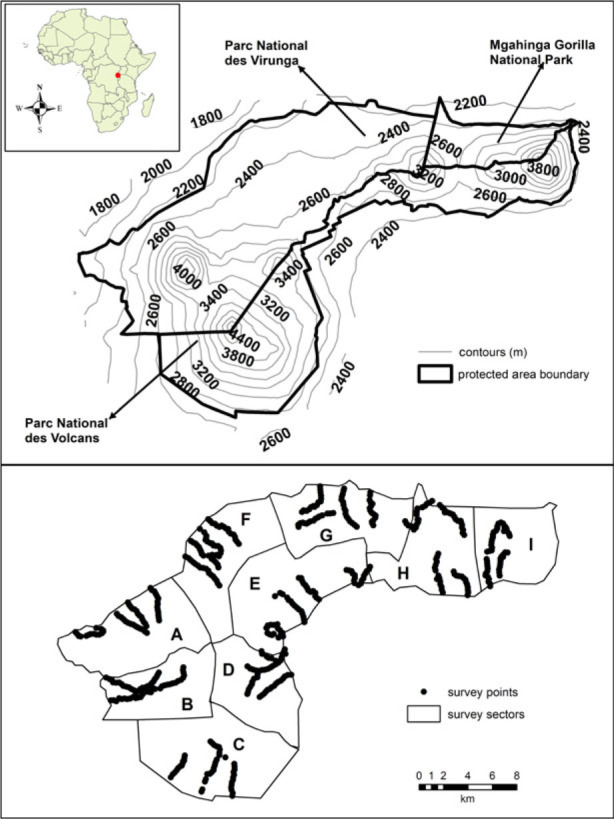
Study area (i.e. Virunga volcanoes) showing the elevation gradient (top) and distance sampling point count transects used to collect the bird data (bottom). The sectors are represented as follows: A = Rubindi, B = Akabarozi, C = Ngando, D = Karisoke, E = Kago, F = Bukima, G = Jomba, H = Kagano - Ntebeko, I = Minoga - Muhavura.

Our study occurred along an elevation gradient (1800–4000 m) in the Virunga volcanoes (1°23'21.56″ S, 29°35'17.29″ E), a montane forest within three east-central African countries: Uganda, Rwanda and the Democratic Republic of Congo (figure 1). The transboundary conservation area (approx. 434 km^2^) consists of Mgahinga Gorilla National Park (MGNP: 33.7 km^2^) in Uganda, Parc National des Volcans (PNV: 160 km^2^) in Rwanda, and Parc National des Virunga (PNVi: 240 km^2^) in the Democratic Republic of Congo. The topography is dominated by six volcanic mountains (Muhavura: 4127 m, Mgahinga: 3474 m, Sabinyo: 3637 m, Visoke: 3711 m, Karisimbi, 4507 m, Mikeno: 4437 m) and the vegetation zones vary largely with increasing elevation [36,37]. The major vegetation types include: alpine/sub-alpine (approx. 3200 m and above), *Hagenia-Hypericum* woodland (approx. 2800–3300 m), bamboo forest (approx. 2500–2800 m), secondary bush/shrub (approx. 1900–2400 m), secondary mixed forest (approx. 1800–2500 m), and mature mixed forest (2400–2600 m) [38,39]. Part of Mgahinga Gorilla National Park was settled briefly by people during the conflicts in Uganda in the 1980s resulting in a cultivated portion of the park that occurs between 1900 and 2400 m becoming secondary habitat. There are also swamp and grassland habitats which occur at multiple elevations.

Climatic conditions are influenced by the mountainous topography with temperature decreasing and precipitation generally increasing with elevation [36,40]. Mean monthly temperatures are fairly stable while there is comparatively higher variability in seasonal rainfall patterns [41]. The region experiences a humid tropical climate characterized by two wet and two dry seasons, with annual rainfall accumulation between 1300 and 2100 mm [42]. While precipitation can be found throughout the year, highest rainfall is observed during the March–May rainy season followed by the September–November rainy season [43].

### (b) Data collection

Species count data were collected according to a distance sampling protocol during the dry season from 10 January through to 2 February 2004 using point count surveys across an elevation gradient. Distance sampling accounts for the imperfect observation of animals with a detection function by establishing a relationship using distance measurements from the survey point to animals [44]. The key assumptions underlying distance sampling are that observations on the point have perfect detection, detection probability decreases with the animal's distance from the point, there is no movement between the observer and the target animal when measuring distances, and that distances to observations are measured without error [44].

The study area was divided into nine sectors to facilitate spatial coverage of the park. Four sectors were located in PNVi (Akabarozi, Rubindi, Bukima, Jomba), three sectors in PNV (Ngando, Karisoke, Kagano) and two sectors (Kagano – Ntebeko, Minoga – Muhavura) were distributed between PNV and MGNP (figure 1). Between three to five transects (1750–4750 m in length) were established in each sector following a stratified sampling (altitude, habitat) approach, with points placed at 250 m intervals along each trail using a hipchain, such that individual sampling points were independent. Survey point transects were walked following a fixed compass direction (e.g. north compass bearing), however in situations where difficult terrain was encountered, paths of least resistance were used. On average, each survey transect consisted of 15 sampling points. A total of 519 sampling points were surveyed across all sectors along an elevation gradient from 1800 to 3900 m with an average of 57 sampling points within each sector and 52 sampling points at each 200 m elevation band. Each sampling point was surveyed on one occasion using a point count protocol [45], during which the observer identified all birds seen or heard for a period of 5 min, after allowing for a 2 min settling period. Bird observations were assigned to one of four distance classes (0–10 m, 10–20 m, 20–50 m, 50–100 m). Observers also recorded elevation and vegetation type (17 categories) at each point. Sampling was carried out in the morning hours (i.e. 6.30 to 11.00). To ensure our single-year surveys were a robust description of the overall bird community in this region, we compared our data to a multi-year dataset collected within a portion of our study region [46] using rank abundance plots. The rank abundance plots revealed close correspondence in bird community patterns, suggesting that our data were sufficient for the task at hand (electronic supplementary material, Appendix S1).

### (c) Variables used to measure the abiotic Grinnelian niche

A pairwise Pearson correlation analysis between elevation and worldclim bioclimatic variables (http://www.worldclim.org) for the study area revealed a strong positive correlation (0.9) with mean annual precipitation and an equally strong negative correlation (−0.95) with mean annual temperature (electronic supplementary material, Appendix S2). Thus, we used elevation as the only covariate in the abundance model, as it is a good proxy for habitat conditions (e.g. climate and vegetation) and is highly correlated with vegetation in the Virunga volcanoes [40], often viewed as a potential niche partitioning gradient for birds [18,20]. We hypothesized that vegetation type might influence the detection of birds during sampling. Accordingly, we combined the 17 vegetation types based on composition and structure into the eight vegetation categories that were then used as covariates in the detection model (see *Hierarchical community model*): alpine/ sub-alpine, *Hagenia-Hypericum* woodland, bamboo forest, secondary bush/shrub, secondary mixed forest, mature mixed forest, grassland and swamp habitat.

### (d) Bird community: variables used to measure the biotic Eltonian niche

The community of birds in the Virunga volcanoes consists of forest interior species, forest generalist species, forest visitors, and species that use other non-forest habitats [47]. A total of 294 bird species across 66 families have been recorded in the Virunga volcanoes including 18 species that are endemic to the Albertine Rift ecoregion [38]. We obtained diet, foraging vertical strata, body size, and activity pattern data from a global database of attributes that describe species' Eltonian niches [48]. The diet and foraging vertical strata data are compositional (i.e. sum to one) and represent the proportion of resource use within a diet category (e.g. nectar, invertebrates, fruit, seed) and the proportion of time spent in five forest strata (i.e. ground, understory, mid-high, canopy, aerial) for each species, respectively. The body size metric is the average weight of each species [49] and activity pattern describes whether a species is nocturnal or diurnal [48].

### (e) Hierarchical community model to estimate abundance

We estimated the abundance of birds along an elevation gradient using a hierarchical community distance sampling model [30,50]. Elevation was used as the only predictor in the abundance model while vegetation classes were used in the observation model to estimate species detection probabilities. Hierarchical community distance sampling models enable the estimation of species and community parameters by linking individual species-specific distance sampling models using community-level normal distributions [30,31]. As a result of information sharing across species, this approach allowed us to include 63 bird species (belonging to 32 families) that were observed with at least 10 observations, compared to only 28 species that we could have analysed using single-species models following standard recommendations that suggest at least 60 observations [44].

We estimated the abundance for each species at a given sampling location by establishing a relationship between detection probability and observed distances to individual birds. We estimated detection probabilities for each species as a function of distance *r* from the sampling point to individual bird observations, assuming a half-normal detection function:

2.1 $$g(r)\;=\exp\;\left(\frac{-r^{2}}{2\sigma_{\mathrm{js}}^{2}}\right),$$

where $\sigma_{\;js}$ is a scale parameter that determines the shape of the detection function at each sampling point *j* for each species *s*. We modelled $\sigma_{\;js}\;$ on a log scale according to:

2.2 $$\log(\sigma_{\;js})=\alpha_{s}+\;\gamma \cdot {\mathrm{veg}}_{j},$$

where *α*_s_ is the species-specific intercept parameter and γ is the effect of vegetation type on detection probability. For the detection model, we used discrete vegetation categories rather than the continuous metric of elevation because we hypothesized that detection was likely to vary by the specific vegetation features of the various habitats. The effect of vegetation on detection was estimated as a fixed effect, which was assumed to be the same across species; however, the overall magnitude in detection probabilities varies by species because the intercept (*α*_s_) is estimated for each species. We modelled the species-specific intercepts (*α*_s_) as random effects, drawn from a community level distribution:

2.3 $$\alpha_{s}∼\;\mathrm{normal}(\mu_{\alpha },\sigma_{\alpha }^{2}),$$

where $\mu_{a}$ is the average intercept parameter across the community (on the log scale) and the variance, $\sigma_{a}^{2}$, represents variability in the intercept across species.

The detection probability for each species is then the expected value, $\bar{P},$ obtained by integrating the detection function *g*(*r*), over all possible realizations of distance *r* [31,44]:

2.4 $$\bar{P}=\int_{0}^{w}g(r)[r^{*}]\;dr,$$

in which $[r^{*}]=2r/w^{2}$ and *w* is the maximum radial distance at which observations were made (i.e. 100 m). In point transect surveys, the probability distribution for distances $\left[r^{*}\right]$ follows a triangular distribution because the area surveyed increases with each distance class and therefore the number of birds available for detection also increases. The probability of detecting a species, $p_{djs,}$ in each distance class (*d* = 1, 2,…..D) with distance breaks $\left[h_{0},h_{1}],[h_{1},h_{2}],\ldots ,[h_{D-1},h_{D}\right]$ at survey point *j* was computed as the integral of *g*(*r*) from the lower break point $h_{d-1}$ to the upper breakpoint *h_d_* adjusting for proportion of area surveyed using the probability distribution of distance r (which we estimated using numerical integration):

2.5 $$p_{\mathrm{djs}}=\frac{2}{w^{2}}\int_{h_{d-1}}^{h_{d}}rg(r)dr.$$

The number of individuals observed within each distance class at sampling point *j* for species *s*, $y_{\;js},$ represents a vector of observations that follows a multinomial distribution:

2.6 $$y_{\;js}∼\mathrm{multinomial}(n_{\;js},p_{\;js}^{c}),$$

where, *n_js_* is the total number of observed individuals at site *j* for species *s*, $\sum_{d}y_{\;js}$, and $p_{\;js}^{c}=p_{djs}/\sum_{d}\;p_{djs}$, is the conditional multinomial cell probability of detecting an individual given that it is in distance class *d*. The total number of observed individuals at *j* for species *s*, $n_{\;js}$, follows a binomial distribution with parameters, $N_{\;js}$ (the true number of individuals of species *s* at sampling point *j*) and $p_{\;js}=\sum_{d}\;p_{djs}$, the probability of observing species *s*:

2.7 $$n_{\;js}∼\;\mathrm{binomial}(N_{\;js},\;p_{\;js}).$$

We estimated abundance *N_js_*, at each site *j* for each species *s*, using a negative binomial distribution (fitted as a Poisson-gamma mixture) such that:

2.8 $$N_{\;js}∼\;\mathrm{Poisson}(\lambda_{\;js}^{*}),$$

in which expected abundance is $\lambda_{\;js}^{*}\;=\lambda_{\;js}.\rho_{\;js}$, where $\rho_{\;js}$ is a gamma-distributed random variable that controls the amount of overdispersion. We included linear and quadratic effects of elevation on estimates of species-specific abundance using a log-link function to account for potential species abundance optima at specific elevation ranges:

2.9 $$\log(\lambda_{\;js})=\beta 0_{s}+\;\beta 1_{s}\cdot {\mathrm{elev}}_{j}+\beta 2_{s}\cdot {\mathrm{elev}}_{j}^{2},$$

where $\beta 0_{s}$ is a species-specific intercept parameter, $\beta 1_{s}$ is the species-specific linear effect of elevation (${\mathrm{elev}}_{j}$), and $\beta 2_{s}$ is the species-specific quadratic effect of elevation. We standardized elevation to have a mean of 0 and standard deviation of 1. To link the species-level parameters at the community level, we assume that the species-specific parameters in the abundance model ($\beta 0_{s}$, $\beta 1_{s}$ and $\beta 2_{s}$) were drawn from community-level normal distributions with hyper-means $\mu_{\beta 0}$, $\mu_{\beta 1}$, $\mu_{\beta 2}$ and hyper-variances $\sigma_{\beta 0}^{2}$, $\sigma_{\beta 1}^{2}$, $\sigma_{\beta 2}^{2}$.

We estimated the parameters in our model using a Bayesian approach with the programs R (R Core Team 2020) and JAGS [51] using the jagsUI package [52]. We ran three parallel chains for 200 000 iterations with a burn-in of 50 000 iterations and thinning rate of 10 to obtain 45 000 posterior samples. We used weakly informative priors for all hyperparameters. Hyper-mean parameters (i.e. $\mu_{\alpha }$, $\mu_{\beta 0},$ $\mu_{\beta 1}$ $\mu_{\beta 2}$) were assumed to come from normal distributions with a mean of 0 and variance of 100. Hyper-variances were assigned a γ prior with shape and scale parameters equal to 0.1. To determine convergence, we visually inspected trace plots and used the potential scale reduction factor (Gelman-Rubin statistic, Rhat), assuming convergence when Rhat < 1.1 [53].

### (f) Multi-dimensional niche calculations

Of the 32 families included in the hierarchical community model, we selected all species from families that had data on two or more species. This resulted in 46 species—60 species pairs—from 15 families. We performed subsequent analyses on species pairs only within the same family to evaluate ecologically plausible associations and improve our interpretation of coexistence mechanisms. We estimated pairwise niche overlap indices along elevation, diet, forest strata, and within-habitat segregation across horizontal space niche dimensions for all 60 species pairs within individual families. For each niche dimension index, we also generated null model expectations based on random species pairs to assess whether species pairs within families showed more or less niche differentiation compared to random (electronic supplementary material, Appendix S3).

#### (i) Environmental habitat gradient (elevation)

We estimated the environmental niche overlap for the 60 species pairs by computing the area of intersection between species abundance-elevation curves as a proportion of the total area for each species’ individual abundance-elevation curve. Using the estimated mean parameter values for $\beta 0_{s}$, $\beta 1_{s}$ and $\beta 2_{s}$, we generated an abundance-elevation response curve across the full range of observed elevation values for each species (following equation (2.9)). For two species A and B, we calculated $N_{\mathrm{AB}}$, the proportion of species A's elevation niche overlapped by species B, using the following equation:

2.10 $$N_{\mathrm{AB}}=\left(\frac{Ov_{\mathrm{AB}}}{T_{A}}\right),$$

where $Ov_{\mathrm{AB}}$ is the area of intersection between species A and species B abundance-elevation response curves and $T_{A}$ is the total area under the curve for species A. We calculated $Ov_{\mathrm{AB}}$ and $T_{A}$ using the approxfun function in R software [54]. We similarly calculated $N_{\mathrm{BA}}$ as the proportion of species B's niche overlapped by species A (note that $Ov_{\mathrm{AB}}=Ov_{\mathrm{BA}}$), resulting in two measures of species overlap for each species pair. The elevation niche overlap index ranges from 0 (entirely different niches) to 1 (complete niche overlap). To enable comparisons of niche overlap indices between abundant and rare species, we standardized species abundance-elevation curves to the same relative scale (maximum abundance = 1) by dividing expected abundance by the maximum value of abundance. Species that had at least 60 observations (i.e. threshold for single species distance sampling model) [44], were considered ‘common’ while species that had between 10 and 59 observations were considered ‘rare’. Following recommendations for interpreting possible niche overlap scenarios in Dormann *et al*. [29], we defined disparate niches as: two species with no niche overlap (i.e. $N_{\mathrm{AB}}\approx N_{\mathrm{BA}}\approx 0$) and partial overlap (i.e. when either $N_{\mathrm{AB}}<0.5$ or $N_{\mathrm{BA}}<0.5$). We categorize two species as having similar niches if both species occupied at least 50% (0.5 niche overlap) of the same elevation gradient (i.e. $N_{\mathrm{AB}}\geq 0.5$ and $N_{\mathrm{BA}}\geq 0.5$). We selected the 50% threshold because it represents the niche overlap value where the effect of species A on B and the effect of species B on A is approximately equal.

#### (ii) Diet and foraging vertical strata

We estimated niche overlap along diet and forging vertical strata niche dimensions, $O_{\mathrm{AB}}$, between species pairs A and B in the same families using Pianka's measure of niche overlap [28,55]:

2.11 $$O_{\mathrm{AB}}=\frac{\sum_{d}^{n}P_{dA}P_{dB}}{\sqrt{\sum_{d}^{n}P_{dA}^{2}\sum_{d}^{n}P_{dB}^{2}}},$$

where $P_{dA}$ and $P_{dB}$ are the proportion of resource use in category *d* along a niche dimension by species A and B, respectively, and *n* is the number of resource categories in a niche dimension. The niche overlap index ranges from 0 (no overlap) to 1 (maximum overlap).

#### (iii) Body size variation

We compared the variation in body sizes for species across families by estimating the coefficient of variation (CV), a ratio of the standard deviation to the mean, a metric that is commonly used for comparing trait variability among and within species [56,57]. Lower body size CV values indicate less variation and diet niche conservatism within a family, while higher body size CV values suggest more variation and low dietary niche overlap.

#### (iv) Spatial site index

The spatial site index, ${\mathrm{SSI}}_{\mathrm{AB}}$, is a measure of within-habitat segregation across horizontal space. It is expressed as a proportion of the total number of sites in which both species are detected (S_AB_) across all the sites in which one or both species was detected during sampling (S_d_):

2.12 $${\mathrm{SSI}}_{\mathrm{AB}}=\left(\frac{S_{\mathrm{AB}}}{S_{d}}\right).$$

Similar approaches have been previously used to estimate spatial overlap (occurrence, territories) between bird species pairs [18,58]. We examined an alternative site index using the latent abundance values from the hierarchical community distance sampling model and assessed detection probability variation among species within families to ensure the spatial site index was robust to detection heterogeneity. Both additional analyses support our approach for computing the within habitat segregation index (electronic supplementary material, Appendix S4).

### (g) An extension: joint species distribution model for warblers

We extended the hierarchical community distance sampling model by estimating a residual correlation matrix between individual species using a joint species distribution modelling framework that directly accounts for imperfect detection [59]. We did this only for the warbler family (Cisticolidae) to evaluate species associations after accounting for abiotic effects for closely related species. Warblers were chosen because they were the only family with both an adequate number of species (five total) and observations across species (i.e. each species was observed at least 79 times). This model is identical to the community model previously described except we removed the gamma distributed over-dispersion parameter and instead incorporated a species-specific site-level random effect arising from a multivariate normal distribution. Specifically, our model for species-specific abundance was thus modified as follows:

2.13 $$\log(\lambda_{\;js})=\beta 0_{s}+\beta 1_{s}\cdot {\mathrm{elev}}_{j}+\;\beta 2_{s}\cdot {\mathrm{elev}}_{j}^{2}+\eta_{\;js},$$

where $\eta_{\;js}$ is a species-specific, site-level random effect drawn from a multivariate normal distribution with an unstructured variance-covariance matrix. We specified a weakly informative inverse Wishart prior for the variance-covariance matrix and estimated the posterior distribution by running three parallel chains for 200 000 iterations with a burn-in of 50 000 iterations and thinning rate of 50 to obtain 9000 posterior samples using R and JAGS. We compared a null model with no covariates to the full model with elevation (equation (2.12)) to evaluate how residual correlations between species abundance estimates change after accounting for environmental variability.

We used the results of our joint species distribution model to validate the interpretation of our two-step approach by examining the general pattern of residual correlations (i.e. joint species distribution model) after accounting for elevational effects. The drawback of using a joint species distribution model alone to study coexistence mechanisms is a decrease in computational efficiency as the number of species pairs increases, and also a lack of clarity in how to interpret pairwise correlations [11,29].

## 3. Results

**Figure 2.**
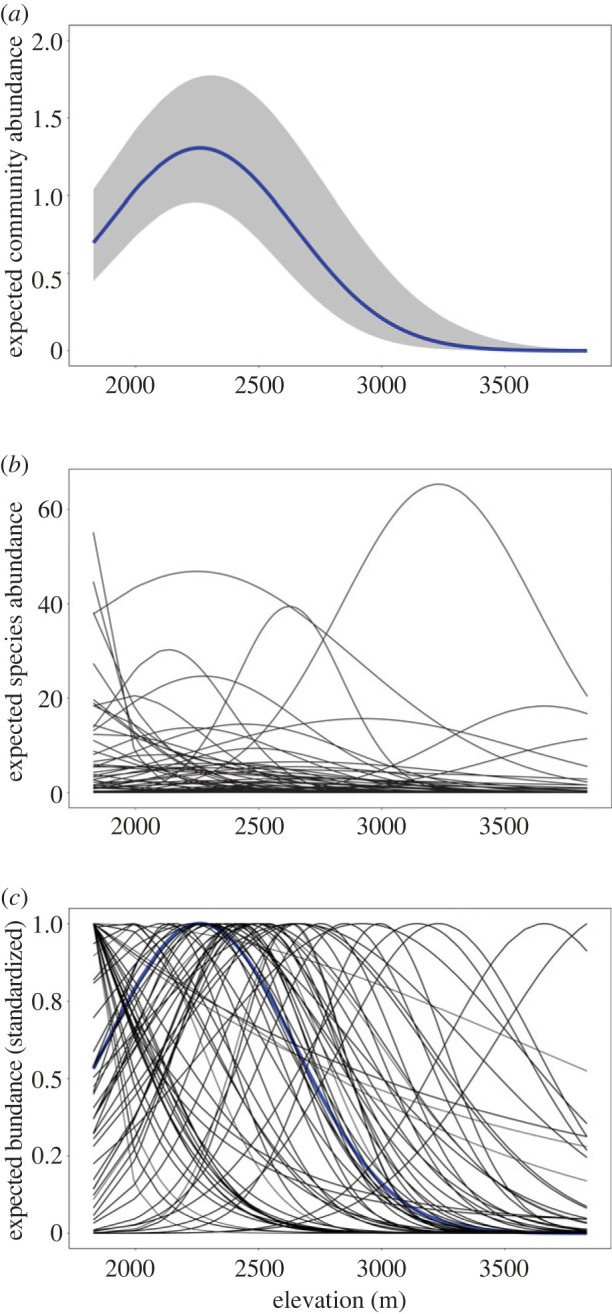
Expected abundance patterns of birds in the Virunga volcanoes in relation to elevation for (*a*) the full community, (*b*) each of the 63 species included in the analysis, and (*c*) each of the species standardized between 0 and 1 (which was done to compare niche overlap indices between rare and abundant species). Expected community level abundance is shown in blue with the 95% credible intervals represented by the grey shading. Species-specific abundance-elevation response curves are shown with thin black lines.

We observed a total of 129 bird species (6039 individuals) within 49 families, including 14 endemic species. Twenty-two per cent of the species (28) had at least 60 records (i.e. common), 27% of the species (35) had between 10 and 59 records (i.e. rare), and 51% of the species (66) had less than 10 records (i.e. very rare and excluded from analyses). Expected community-level mean abundance showed a significant relationship with elevation (figure 2*a*) across the 63 bird species (from 32 families) that were included in our analysis (electronic supplementary material, Appendix S5). On average, expected abundance increased with elevation up to approximately 2200–2400 m and then decreased (posterior mean of $\mu_{\beta 1}$: −0.99, 95% confidence interval (CI): −1.37, −0.62; $\mu_{\beta 2}$: −0.45, 95% CI: −0.6, −0.31, on a log scale) (figure 2*a*; electronic supplementary material, Appendix S6). The peak in which average community abundance was maximized is representative of secondary habitat with an approximate average annual temperature of 15°C. Species-specific abundance patterns showed marked variation in relation to elevation (figure 2*b,c*). Expected species abundance curves were characterized by three patterns: a mid-elevation peak in abundance (51%) ranging between approx. 2000–2800 m, decreasing with increasing elevation (38%, peak: 1800–2100 m), and increasing with increasing elevation (the remaining 11%, peak: 2700–3800 m). Detection probability was highest in grassland vegetation, followed by swamp, alpine/sub-alpine, *Hagenia-Hypericum* woodland, bamboo, secondary bush/shrub, secondary mixed forest and lastly, mixed forest (electronic supplementary material, Appendix S7).

### (a) Multidimensional niche

#### (i) Environmental habitat gradient (elevation)

**Figure 3.**
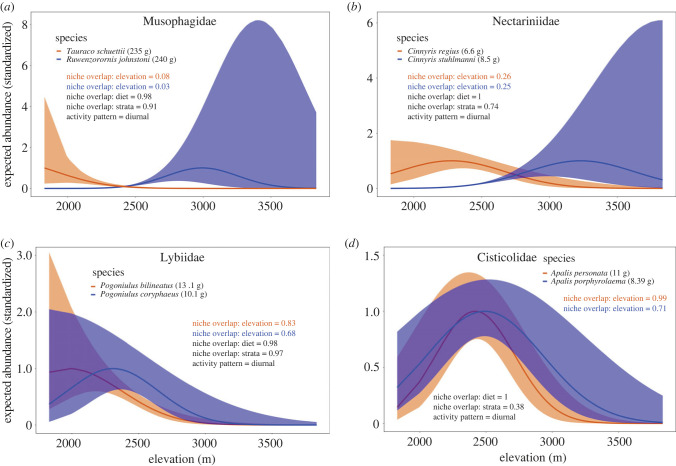
Standardized species abundance curves (maximum expected value = 1) in relation to elevation in the Virunga volcanoes for four bird species pairs across four families: (*a*) Musophagidae, (*b*) Nectariniidae, (*c*) Lybiidae and (*d*) Cisticolidae. The four pairs illustrate examples of (*a*) no niche overlap, (*b*) partial niche overlap, and (*c*) and (*d*) similar niches with respect to an environmental habitat gradient (characterized by elevation). Expected species abundance are shown with thick blue and orange lines; light blue and orange background shading show the 95% credible intervals.

The estimated elevation overlap indices revealed disparate co-abundance patterns (i.e. no niche overlap or partial overlap) for 33 species pairs (55%) within 10 families and similar co-abundance patterns for the remaining species pairs within 12 families (table 1; electronic supplementary material, Appendix S8). Four species pairs had no elevation niche overlap (e.g. *Tauraco schuettii* and *Ruwenzorornis johnstoni;* figure 3*a*), 29 species pairs showed partial elevation overlap (e.g. *Cinnyris regius* and *Cinnyris stuhlmanni*; figure 3*b*), and 27 species pairs had similar elevation niches (e.g. *Pogoniulus bilineatus* and *Pogoniulus coryphaeus; Apalis personata and Apalis porphyrolaema*; figure 3*c,d*). Null distribution analyses revealed substantially more elevational niche differentiation for species pairs within families than would be expected at random (i.e. only 22% of randomly selected pairs expected to have disparate co-abundance patterns; electronic supplementary material, Appendix S3).

**Table 1.** Estimated niche overlap indices for 60 bird species pairs along an environmental habitat gradient (elevation), diet, foraging vertical strata (strata), and within habitat segregation across horizontal space (spatial site index) in the Virunga volcanoes. (All species were active during the day, indicating no niche partitioning along a coarse temporal dimension. Body mass (grams) is shown in parentheses next to each species name. Potential niche partitioning refers to the most likely mechanisms of partitioning based on model results. Full species names are provided in the electronic supplementary material, Appendix S5.)

family	species pairs		Pianka niche overlap				spatial site index	potential niche partitioning
			elevation		symmetrical			
	species A (g)	species B (g)	species B to A	species A to B	diet	strata		
Musophagidae	*T. schuetti* (235)	*R. johnstoni* (240)	0.08	0.03	0.98	0.91	0.00	elev
Nectariniidae	*C. regius* (6.58)	*C. stuhlmanni* (8.5)	0.25	0.26	1.00	0.93	0.06	elev
Nectariniidae	*C. regius* (6.58)	*C. venustus* (6.56)	1.00	0.80	1.00	0.80	0.04	spatial
Nectariniidae	*C. regius* (6.58)	*C. alinae* (12.56)	0.86	0.24	0.98	1.00	0.02	elev
Nectariniidae	*C. regius* (6.58)	*H. collaris* (6.98)	0.81	0.12	0.64	0.92	0.02	elev
Nectariniidae	*C. regius* (6.58)	*N. johnstoni* (15.16)	0.13	0.10	1.00	0.83	0.00	elev
Nectariniidae	*C. stuhlmanni* (8.5)	*C. venustus* (6.56)	0.21	0.16	1.00	0.74	0.02	elev
Nectariniidae	*C. stuhlmanni* (8.5)	*C. alinae* (12.56)	0.14	0.04	0.98	0.93	0.02	elev
Nectariniidae	*C. stuhlmanni* (8.5)	*H. collaris* (6.98)	0.11	0.01	0.64	0.94	0.00	elev
Nectariniidae	*C. stuhlmanni* (8.5)	*N. johnstoni* (15.16)	0.78	0.57	1.00	0.88	0.12	spatial
Nectariniidae	*C. venustus* (6.56)	*C. alinae* (12.56)	0.79	0.27	0.98	0.80	0.00	elev
Nectariniidae	*C. venustus* (6.56)	*H. collaris* (6.98)	0.68	0.13	0.64	0.92	0.00	elev
Nectariniidae	*C. venustus* (6.56)	*N. johnstoni* (15.16)	0.06	0.06	1.00	0.82	0.00	elev
Nectariniidae	*C. alinae* (12.56)	*H. collaris* (6.98)	1.11	0.60	0.75	0.92	0.00	spatial
Nectariniidae	*C. alinae* (12.56)	*N. johnstoni* (15.16)	0.02	0.04	0.98	0.83	0.00	elev
Nectariniidae	*H. collaris* (6.98)	*N. johnstoni* (15.16)	0.01	0.03	0.64	0.89	0.00	elev
Lybiidae	*P. bilineatus* (13.1)	*P. coryphaeus* (10.7)	0.68	0.83	0.98	0.94	0.10	spatial
Lybiidae	*P. bilineatus* (13.1)	*T. purpuratus* (76.1)	0.98	0.49	0.62	0.50	0.15	elev, strata
Lybiidae	*P. coryphaeus* (10.7)	*T. purpuratus* (76.1)	0.81	0.33	0.72	0.62	0.05	elev
Cisticolidae	*A. porphyrolaema* (8.39)	*A. personata* (11)	0.71	1.00	1.00	0.38	0.15	strata, spatial
Cisticolidae	*A. personata* (11)	*C. chubbi* (16.03)	0.85	0.98	1.00	0.24	0.12	strata, spatial
Cisticolidae	*A. personata* (11)	*O. ruwenzorii* (9.9)	0.74	0.80	1.00	0.47	0.14	strata, spatial
Cisticolidae	*A. personata* (11)	*P. bairdii* (13.4)	0.62	0.23	1.00	0.00	0.05	elev, strata
Cisticolidae	*A. porphyrolaema* (8.39)	*C. chubbi* (16.03)	1.00	0.83	1.00	0.00	0.24	strata, spatial
Cisticolidae	*A. porphyrolaema* (8.39)	*O. ruwenzorii* (9.9)	0.81	0.62	1.00	0.09	0.19	strata, spatial
Cisticolidae	*A. porphyrolaema* (8.39)	*P. bairdii* (13.4)	0.75	0.20	1.00	0.92	0.07	elev
Cisticolidae	*C. chubbi* (16.03)	*O. ruwenzorii* (9.9)	0.74	0.68	1.00	0.97	0.27	spatial
Cisticolidae	*C. chubbi* (16.03)	*P. bairdii* (13.4)	0.63	0.21	1.00	0.00	0.08	elev, strata
Cisticolidae	*O. ruwenzorii* (9.9)	*P. bairdii* (13.4)	0.95	0.34	1.00	0.00	0.14	elev, strata
Phylloscopidae	*P. laetus* (9.54)	*P. umbrovirens* (8.6)	0.23	0.12	1.00	0.58	0.03	elev, strata
Malaconotidae	*D. gambensis* (31.9)	*L. aethiopicus* (49.44)	0.56	0.12	0.75	0.13	0.00	elev, strata
Malaconotidae	*D. gambensis* (31.9)	*L. luehderi* (42.9)	0.43	0.05	0.99	0.00	0.03	elev, strata
Malaconotidae	*D. gambensis* (31.9)	*L. poensis* (44.9)	0.63	1.00	0.99	0.31	0.12	strata, spatial
Malaconotidae	*L. aethiopicus* (49.44)	*L. luehderi* (42.9)	1.22	0.74	0.76	0.94	0.05	spatial
Malaconotidae	*L. aethiopicus* (49.44)	*L. poensis* (44.9)	0.10	0.79	0.79	0.89	0.01	elev
Malaconotidae	*L. luehderi* (42.9)	*L. poensis* (44.9)	0.06	0.75	0.99	0.94	0.01	elev
Malaconotidae	*D. gambensis* (31.9)	*T. dohertyi* (35.02)	0.72	0.78	0.99	0.00	0.01	strata, spatial
Malaconotidae	*L. aethiopicus* (49.44)	*T. dohertyi* (35.02)	0.07	0.35	0.76	0.94	0.04	spatial
Malaconotidae	*L. luehderi* (42.9)	*T. dohertyi* (35.02)	0.03	0.30	1.00	1.00	0.00	spatial
Malaconotidae	*L. poensis* (44.9)	*T. dohertyi* (35.02)	0.97	0.66	0.99	0.94	0.05	spatial
Cuculidae	*C. monachus (201.31)*	*C. klaas* (27.37)	0.86	0.64	0.69	0.54	0.00	strata, spatial
Cuculidae	*C. monachus* (201.31)	*C. solitarius* (76.73)	0.87	0.37	0.74	1.00	0.00	spatial
Cuculidae	*C. klaas* (27.37)	*C. solitarius* (76.73)	1.00	0.57	0.98	0.54	0.00	strata, spatial
Pycnonotidae	*E. latirostris* (26.2)	*A. nigriceps* (32.8)	0.65	0.99	0.93	0.86	0.27	spatial
Pycnonotidae	*E. latirostris* (26.2)	*P. barbatus* (32.1)	0.83	0.90	0.83	0.96	0.23	spatial
Pycnonotidae	*A. nigriceps* (32.8)	*P. barbatus* (32.1)	0.91	0.65	0.92	0.85	0.16	spatial
Columbidae	*C. arquatrix* (400)	*S. lugens* (155)	0.96	0.52	0.78	0.15	0.06	strata, spatial
Columbidae	*C. arquatrix* (400)	*S. semitorquata* (176)	0.96	0.47	0.67	0.25	0.00	strata, spatial
Columbidae	*S. lugens* (155)	*S. semitorquata* (176)	1.00	0.90	0.97	0.98	0.02	spatial
Platysteiridae	*Batis diops* (12.7)	*Batis molitor* (11.64)	1.00	0.93	1.00	0.09	0.03	strata, spatial
Ploceidae	*P. alienus* (22.2)	*P. baglafecht* (31.6)	0.64	0.88	0.96	0.55	0.00	strata, spatial
Fringillidae	*C. frontalis* (12.3)	*C. gularis* (16)	0.97	1.00	0.70	0.82	0.05	spatial
Estrildidae	*E. astrild* (8.29)	*E. kandti* (7.48)	0.24	0.28	1.00	0.94	0.13	spatial
Acrocephalidae	*I. natalensis* (11.7)	*I. similis* (11.1)	0.62	1.00	1.00	1.00	0.03	spatial
Muscicapidae	*M. fisheri* (23.4)	*B. comitatus* (14.1)	0.36	0.89	1.00	0.00	0.00	strata, spatial
Muscicapidae	*M. fisheri* (23.4)	*C. archeri* (22.8)	0.29	0.92	1.00	0.71	0.01	spatial
Muscicapidae	*M. fisheri* (23.4)	*P. stellata* (18.6)	0.38	0.58	0.95	0.55	0.00	strata, spatial
Muscicapidae	*B. comitatus* (14.1)	*C. archeri* (22.8)	0.77	0.99	1.00	0.65	0.02	spatial
Muscicapidae	*B. comitatus* (14.1)	*P. stellata* (18.6)	1.00	0.62	0.95	0.82	0.04	spatial
Muscicapidae	*C. archeri* (22.8)	*P. stellata* (18.6)	1.00	0.48	0.95	0.9	0.04	elev

#### (ii) Within-habitat segregation

The spatial site indices showed that all species pairs had very low observed co-occurrence with an average of 6% (s.d. = 7.3%) across the sites in which at least one of the species was observed (range: 0–27%; table 1). The average value was slightly higher for the 27 species pairs with similar elevation niches, which were observed to co-occur at 9.3% of sites on average (s.d. = 8.8%, range: 0–27%). Additional analyses revealed that the low number of co-occurrence observations at survey sites were not simply a result of differences in detection probability across species. Instead, we found that species within the same families had more similar detection probabilities than across families (expected scale parameter ($\sigma_{\mathrm{js}}$) was 0.48 for random species pairs versus 0.24 for pairs within families; electronic supplementary material, Appendix S4).

#### (iii) Foraging forest strata, diet, body size, and activity pattern

We found moderate support for community structuring across vertical foraging strata and less support for stratification across diet (table 1; electronic supplementary material, Appendix S3). Twenty-two species pairs (37%) had low forest strata niche overlap (i.e. ≤ 0.6 on the Pianka scale; [57]), which was significantly fewer species than would be expected at random (49% of pairs with weak forest strata niche partitioning expected based on null distribution; electronic supplementary material, Appendix S3). Of the 22 species pairs with low forest strata niche overlap, 55% (12 pairs) had similar elevation niches suggesting that partitioning along the forest strata niche dimension is another important mechanism promoting coexistence of birds in the study area. However, the expected number of species pairs with low forest strata niche overlap from a null distribution analysis (49%) was higher than what we found in the observed community (37%; electronic supplementary material, Appendix S3), indicating relative niche conservatism along the vertical forest strata axis among species within families compared to random species pairs across families. Diet niche overlap was high (≥ 0.6) for all 60 species pairs, and as such much more prevalent than expected at random (44%; electronic supplementary material, Appendix S3). For those species' pairs with similar elevation niche indices, the diet niche overlap indices were even higher (average = 0.93, CV = 11%) compared to species with disparate elevation patterns (average = 0.9, CV = 17%). Similarly, all species were categorized as active during the day and thus there is no evidence of temporal niche partitioning among these species. We found high variation in the average body sizes among species pairs (i.e. CV ≥25%) for a third of the families (although species within families tended to have more similar body sizes than across families; electronic supplementary material, Appendix S3): Lybiidae (three species; CV = 111%), Cuculidae (three species; CV = 88%), Columbidae (three species; CV = 54%), Nectariniidae (six species; CV = 39%), Cisticoladae (five species; CV = 26%), revealing another potential mechanism for community structuring based on food exploitation and territoriality. However, because we did not have body size data for individuals within our study, it was impossible to assess this quantitatively.

### (b) Residual correlations: (Cisticolidae)

**Figure 4.**
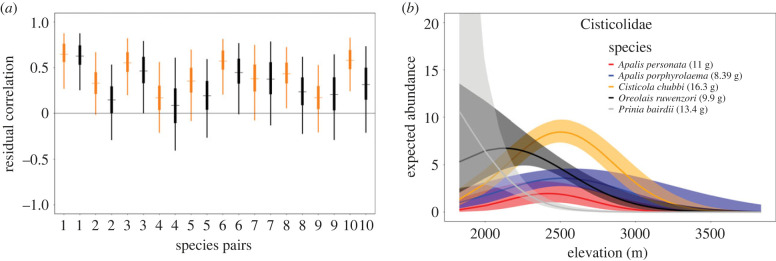
Results from the warbler (Cisticolidae) community model extension used to estimate residual correlations among species pairs in the Virunga volcanoes. (*a*) Pairwise correlations in residual abundance of the five warbler species using an intercept-only abundance model (orange) and a model with linear and quadratic effects of elevation (black). The horizontal line shows no correlation. Mean values are shown by the thin horizontal line with 50% (thick) and 95% (thin) credible intervals shown with vertical bars. (*b*) Expected species abundance curves in relation to elevation for the five warbler species (non-standarized). Mean values are shown with solid lines and 95% credible intervals are represented by background shading. Species pair 1 = (*Apalis personata* and *Apalis porphyrolaema*), species pair 2 = (*Apalis personata* and *Cisticola chubbi*), species pair 3 = (*Apalis personata* and *Oreolais ruwenzorii*), species pair 4 = (*Apalis personata* and *Prinia bairdii*), species pair 5 = (*Apalis porphyrolaema* and *Cisticola chubbi*), species pair 6 (*Apalis porphyrolaema* and *Oreolais ruwenzorii*), species pair 7 = (*Apalis porphyrolaema* and *Prinia bairdii*), species pair 8 = (*Cisticola chubbi* and *Oreolais ruwenzorii*), species pair 9 = (*Cisticola chubbi* and *Prinia bairdii*), species pair 10 = (*Prinia bairdii* and *Oreolais ruwenzorii*).

Using our modified hierarchical community distance sampling model that estimated residual pairwise correlations between species, we found a decrease in positive residual correlations after accounting for elevation as compared to the null model (figure 4; electronic supplementary material, Appendix S9), providing further support that the abiotic environment (measured through an elevational habitat gradient) is important for niche partitioning across species within this family. For the null model, residual correlations among the five warbler species were mostly positive (95% CIs for 7 of the 10 species pairs did not overlap zero). After accounting for elevation, all residual correlations decreased, with correlations of three species pairs being significantly different from zero (i.e. 95% CI not overlapping zero). For example, the residual correlations between species *Apalis personata* and *Cisticola chubbi* (figure 4*a,* species pair 2) were positive in the null model (0.37, 95% CI 0.02–0.69) but overlapped zero in the covariate model (0.15, 95% CI −0.27–0.52).

## 4. Discussion

We estimated niche overlap indices across abiotic Grinnellian and biotic Eltonian niche dimensions to evaluate how ecologically similar bird species can coexist within a species-rich community, and to tease apart how the underlying coexistence mechanisms relate to interspecific associations. We found strong support for environmental habitat partitioning across an elevation gradient as an important mechanism of species coexistence, with 55% of species pairs having separate elevation niches (table 1). For the remaining species pairs, within-habitat segregation across horizontal space and to a lesser extent vertical stratification across foraging strata were the most likely mechanisms of species coexistence, with limited support for niche partitioning across diet and activity patterns. Quantifying niche overlap indices along multiple niche dimensions provides a mechanistic understanding of community structuring [28,60]. Together, our results suggest partitioning across multiple levels of spatial organization is a key mechanism that can give rise to the stable coexistence of closely related species in diverse communities.

Species stratification by an environmental elevation gradient was an important mechanism in determining co-abundance patterns of sympatric bird species. Expected abundance peaked around 2200–2400 m on average across the community, with marked variation in species specific optima (ranging between 1800–3850 m; figure 2; electronic supplementary material, Appendix S8). Vegetation productivity in the Virunga region peaks between 2000–2400 m [40], which could potentially explain the high expected abundance at the community level. Elevation niche partitioning was identified as a potential coexistence mechanism in over half of the species pairs in the community. Together with the estimated reduction in residual pairwise correlations in the extended warbler model (figure 4), these results provide strong evidence of niche partitioning along this Grinellian mechanism for ecologically similar bird species in the Virunga volcanoes, consistent with previous studies in the Cameroonian mountains [61], Himalayan highlands [26], tropical Andes [62,63] and in New Guinea highlands [64,65]. A recent survey of birds covering a third of the study area (Parc National des Volcans in Rwanda) identified 57% of species to be associated with narrow elevation bands (less than 300 m; [66]).

After accounting for niche partitioning across the abiotic environmental elevation gradient, vertical foraging stratification and within habitat horizontal stratification served as important mechanisms of coexistence. Species pairs with similar elevation niches were observed to co-occur on average at only 9.3% of the sites where at least one member of the species pair was present, representing substantial within habitat segregation across horizontal space (table 1), and nearly half (44%) of those species’ pairs had low to moderate forest strata overlap (≤ 0.6). These results imply that species niches are partitioned across multiple spatial dimensions, highlighting the importance of multi-dimensional and multi-scale space use when estimating ecological niches. We suspect that the co-occurrence patterns for species pairs are likely to be influenced by diffuse competition [67,68], and an analysis estimating the occurrence of one species conditional on all species observed at a site would probably provide different results [4]. Interestingly, the proportion of species with low to moderate forest strata overlap was higher for random species pairs in a null distribution analysis compared to species pairs within families (electronic supplementary material, Appendix S3). These results suggest that there is niche conservatism along the forest strata niche dimension within closely related species pairs, which could be explained by species pairs within families having similar diets. Nevertheless, 44% of species pairs within families that had similar elevation niches had low forest strata niche overlap. We found less support for niche partitioning based on diet, with all species pairs having substantial overlap in niche indices (greater than 0.6; table 1). However, these indices were calculated using broad resource categories, which did not allow us to assess how variation in diet preferences and resource availability (e.g. insects) over space and time influence species coexistence. Fine scale data for the time of day when bird species are active would probably reveal greater niche partitioning along temporal dimensions of diet. A comparison of the observed diet niche overlap indices for species pairs within families to null model distributions reveals that diet is phylogenetically conserved within families (electronic supplementary material, Appendix S3). We found some variation in body sizes among species pairs within families (table 1; electronic supplementary material, Appendix S3), which could help enable species to exploit different diet items when co-existing with other species that have similar diet preferences. Variation in resource availability has been found to be an essential mechanism of co-existence in three sympatric turacos (*Corythaeola cristata* (1000 g), *Tauraco schuetti* (235 g), *Ruwenzorornis johnstoni* (240 g)) in Nyungwe national park [69]. At sites where the three species co-occurred, variation in resource availability over time and diversity in diet preferences enabled coexistence. Although all species included in our analyses were categorized as active during the day, additional partitioning could occur as a result of variation in species activity patterns (e.g. foraging strategies) at fine temporal scales throughout the day [23], or as a result of interspecific territoriality [24]. We were unable to explore such mechanisms using the available data.

While our analyses highlight that coexistence of ecologically similar species is facilitated by variation in vertical and horizontal space use, and to a lesser extent variation in diet preferences (table 1), we were unable to quantify how coexistence mechanisms vary across space [26] and time [69]. For example, consider two species (*Cinnyris regius* and *Cinnyris stuhlmanni*) with partial elevation overlap (figure 3*b*): vertical forest strata niche partitioning is likely to be more important for individuals of both species within the overlapping environmental niche space compared to individuals at species optima or edges of the gradient. Further, coexistence mechanisms are probably influenced by spatio-temporal variations in resource availability, such that at locations/times with high resource availability species can coexist, while at locations/times of low resource availability one species may exclude the other. Incorporating such mechanisms into joint species distribution models is an important avenue to further understand structuring of ecological communities but also requires different types of data than those that are typically collected in standard point counts.

Our point count data were collected from a single year with relatively short-duration surveys. While studies have shown that such short-duration point counts can be sufficient to characterize bird communities [70,71], longer point count durations implemented over multiple years have the potential to increase detections of rare species [70,72] and to capture fine scale niche dynamics that are seasonally dependent.

The 63 species included in our models contained a similar proportion of forest specialists (67% versus 64% of species) and generalists (30% versus 32%) as compared with the 129 total species observed during sampling (electronic supplementary material, Appendix S10; [73]). This suggests that our niche overlap results, which only included species that were detected with at least 10 observations, are likely to be representative of the community. However, it is certainly possible that there are unmeasured differences among and between the more common and the more rare species. Collecting long-term species abundance data has the potential to increase the sample sizes for those rare and elusive species and would also allow for analyses of the dynamic processes that influence species coexistence mechanisms seasonally and annually.

## 5. Conclusion

Our study provides important insights into coexistence mechanisms of ecologically similar sympatric bird species within a highly diverse community. Coexistence patterns were largely determined by spatial niche partitioning expressed via an environmental elevation gradient (abiotic) and within-habitat segregation across horizontal space and vertical forest strata (biotic). In this diverse bird community, abiotic and biotic factors combine at both broad and fine scales to promote coexistence, well beyond that which can be explained by chance alone**.**

## Data Availability

Data are available from the Dryad Digital Repository at: https://doi.org/doi:10.5061/dryad.fttdz08z8 [74] and code are hosted by Zenodo at: https://doi.org/10.5281/zenodo.7951611 [75]. Additional information is provided in the electronic supplementary material [76].
